# Light‐Driven Phase Engineering in 2D Materials From Mechanistic Assignment to Ambient Near‐Field Control

**DOI:** 10.1002/smll.76269

**Published:** 2026-10-13

**Authors:** Gour Mohan Das, Mika Pettersson

**Affiliations:** ^1^ Nanoscience Center, Department of Chemistry University of Jyväskylä Finland

**Keywords:** 2D materials, light‐driven phase transitions, light‐induced transformations, near‐field excitation, s‐SNOM

## Abstract

Light‐driven phase transitions and transformations provide a route for post‐synthesis control of structure, charge order, ferroic polarization, chemistry, and local transport in 2D materials. Optical excitation can activate carrier dynamics, phonon coupling, heating, strain redistribution, vacancy formation, surface reactions, and substrate‐mediated effects during one experiment. Mechanistic assignment consequently requires separate identification of the process initiating conversion and the mechanism preserving the modified material. We organize representative experiments into seven practical classes based on the dominant experimentally supported mechanism and outline experimental criteria for distinguishing competing contributions. A quantitative workflow combines independent material verification, controlled perturbations, temporal measurements, and material‐specific descriptors of conversion and stabilization. Far‐field laser processing remains valuable for patterning and device fabrication, although diffraction‐limited illumination averages over nanoscale heterogeneity. Tip‐enhanced femtosecond excitation in scattering‐type scanning near‐field optical microscopy provides a complementary route by localizing excitation and linking nanoscale writing with local optical, structural, and chemical verification. Localized excitation can support reproducible ambient nanoscale phase control when excitation, phase identity, and retention are verified within one region.

## Graphical abstract

Light‐driven phase engineering in 2D materials centers on identifying what initiates conversion and what stabilizes the resulting phase. A practical workflow combines material verification, controlled driver tests, temporal measurements, and retention analysis. Tip‐enhanced near‐field excitation extends the strategy toward ambient nanoscale writing with local optical, structural, and chemical verification.

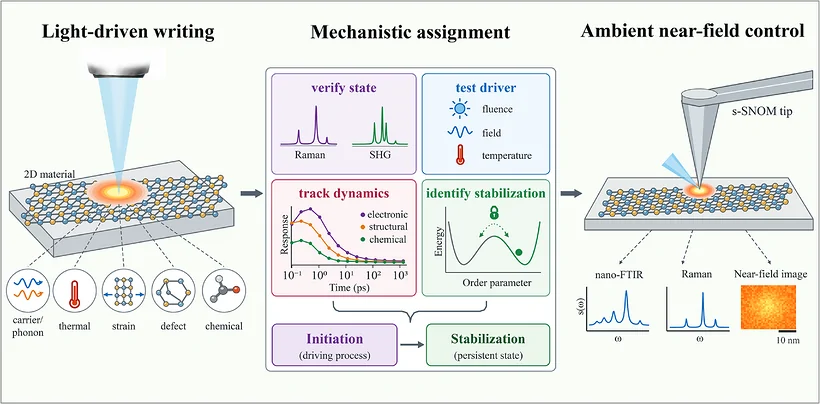

## 1 Introduction

Phase transitions provide a direct way to change the function of a material [1]. Changes in lattice structure, charge order, magnetic order, or band topology can strongly modify the electronic, optical, magnetic, and catalytic properties of 2D materials [2, 3, 4]. Phase transitions are fundamental events in condensed matter physics and useful tools for materials design, device engineering, and nanoscale patterning [5, 6]. 2D materials are especially attractive for phase control because the 2D limit makes structure, charge, strain, dielectric screening, and environmental coupling strongly interdependent. Many layered compounds, including transition‐metal dichalcogenides, charge‐density‐wave materials, layered ferroelectrics, and layered topological materials, host competing phases separated by small energy differences [1, 6, 7]. Their phase stability can be modified by thickness, substrate interaction, strain, electrostatic doping, chemical potential, defects, and local stoichiometry. The sensitivity makes phase engineering in 2D materials promising for electronics, memory, ferroic devices, and emerging quantum technologies [6, 7, 8].

Light adds a practical stimulus for phase control. It can be applied after synthesis, directed to selected regions, and delivered from continuous‐wave illumination to femtosecond pulses. Depending on the material and excitation conditions, photons can create nonequilibrium carriers, drive electron‐phonon coupling, launch coherent lattice motion, raise the local temperature, or induce chemical and structural modifications [9, 10, 11, 12, 13]. Phase‐engineered MoS_2_ contacts demonstrate the device value of locally patterned structural polymorphs [14]. Laser irradiation can also switch correlated electronic states via thermal‐history‐dependent processes in V_2_O_3_ thin films [15]. In 2D materials, optical processing can drive structural conversion, thinning, oxidation, and local patterning [6, 16]. These examples show that light can actively change material properties after synthesis and can also probe phase behavior. In the discussion below, a phase transition refers to conversion between distinct structural, electronic, ferroic, magnetic, or charge‐ordered states of a material. The broader term light‐induced transformation includes persistent or metastable modifications such as oxidation, thinning, defect formation, compositional change, or nanostructuring that can occur without forming a distinct material phase. Such transformations are included because they can occur during the same optical exposure as a phase transition and can generate similar Raman, optical, electrical, or topographic signatures. Separating genuine phase conversion from accompanying material modification forms part of the mechanistic assignment considered throughout the Perspective.

The same versatility creates the main interpretive problem. In 2D materials, optical excitation rarely acts on a single degree of freedom. During one exposure, carriers can be generated, phonons excited, heat accumulated, strain redistributed, vacancies created, adsorbates removed, oxidation promoted, and substrate coupling modified [17, 18]. The observed modification may arise from an intrinsic electronic or lattice transition, thermal conversion, strain relaxation, vacancy stabilization, chemical modification, or coupled processes [19, 20, 21, 22]. The ambiguity becomes especially important under ambient conditions, where oxygen, moisture, residues, substrate adhesion, heat flow, strain gradients, and defect history are part of the experiment. A light‐written region should be described by the process initiating the change and the physical or chemical process stabilizing the final phase.

Far‐field laser irradiation remains the most common optical method for phase engineering because it is simple, flexible, and compatible with standard optical microscopy. It has enabled local modification in MoTe_2_, MoS_2_, graphene, black phosphorus, and other 2D systems [18, 23]. Its main limitation is spatial averaging. The optical spot is usually diffraction‐limited and much larger than nanoscale variations in thickness, wrinkles, grain boundaries, vacancies, adsorbates, residues, and substrate contact. Within the same irradiated region, different sites may cross different thresholds for heating, strain relaxation, defect formation, or chemical reaction. Spatial heterogeneity helps explain why nominally similar laser powers and exposure times can produce different outcomes across flakes, substrates, and laboratories. Femtosecond laser exposure of MoTe_2_ can provide nonlinear optical imaging, layer‐by‐layer thinning, 2H→1T′ conversion, and subwavelength nanoripple formation as the laser fluence is increased [21]. Laser‐patterned MoTe_2_ homojunctions can form stable metallic contact regions, with Te vacancies triggering the local 2H→1T′ transition [20]. In In_2_Se_3_, focused laser exposure can drive the *β*′ →*α* transition by laser‐induced wrinkling and manipulation of accumulated internal strain [19]. These examples show that successful light‐assisted phase control can arise from more than one mechanism. The optical stimulus and the stabilizing process must be identified separately.

This Perspective examines mechanistic assignment in light‐driven phase transitions and transformations of 2D materials, with emphasis on ambient processing and nanoscale optical control. The discussion first considers how interfaces, strain, defects, and environment influence phase stability in atomically thin materials. Seven practical classes then organize representative transformations according to the dominant experimentally supported initiation and stabilization mechanisms. A dedicated experimental workflow connects independent material verification with controlled perturbations, temporal measurements, and quantitative descriptors. The final sections compare controlled and ambient excitation, identify limitations of far‐field writing, and examine tip‐enhanced femtosecond excitation in s‐SNOM as a route toward verifiable nanoscale phase control.

## 2 Phase Transitions in 2D Materials

Phase stability in a 2D material reflects the balance among lattice symmetry, electronic order, strain, charge density, defects, and interfaces. In bulk crystals, many of these variables can often be treated separately. In atomically thin materials, they are strongly connected because the whole crystal is close to a surface or interface. A substrate can stabilize one phase over another, a small amount of strain can change the transition barrier, and a local defect or edge can act as a nucleation site. Phase identity can vary locally across a nominally uniform atomically thin 2D flake [1, 6, 24]. In a monolayer or few‐layer crystal, almost every atom lies close to a surface or substrate. Interfaces play a major role in deciding which phase is stable. A substrate can impose strain, screen charge interactions, modify heat flow, transfer charge, or pin domains [1, 25, 26]. Adsorbates, residues, wrinkles, grain boundaries, and vacancies can further change the local energy balance [1, 6, 27]. Effects that are secondary in a bulk crystal can decide where a transition begins and which phase remains after the stimulus is removed in a 2D flake. This local sensitivity makes phase engineering powerful and also demands careful structural and chemical verification.

A further distinction is between equilibrium and metastable phases. A written region may persist because it is thermodynamically favored under the experimental conditions, or because it is trapped behind an energy barrier [1, 6, 28]. In 2D materials, such trapping can be strengthened by substrate adhesion, strain pinning, finite domain size, vacancies, or chemical modification [1, 6]. Persistent optical contrast, Raman changes, or altered conductance identify a modified material. Independent structural or chemical evidence may still be required to verify its phase identity [6, 20, 28]. The distinction is essential for light‐driven experiments, where the observed phase can be stabilized by a process different from the optical event that initiated it. The application value of light‐driven phase engineering follows directly from this strong local tunability. A single 2D material can contain metallic contacts, semiconducting channels, ferroic domains, optically modified regions, or chemically active interfaces after local writing [6, 18]. Table 1 summarizes representative application directions and shows why mechanistic assignment matters for phase‐written 2D materials instead of attempting a complete device review. Figure 1 summarizes the interacting responses triggered by optical excitation and the challenge of distinguishing initiation from stabilization.

**TABLE 1:** Application relevance of light‐induced phase transitions and transformations in 2D materials.

Application direction	What laser‐induced modification enables	Device / functional relevance
Low‐resistance contacts and heterophase junctions [20]	Local conversion of a semiconducting phase into a metallic phase, such as 2H→1T′ MoTe_2_	Addresses contact resistance in 2D transistors and enables in‐plane metallic/semiconducting junctions
Local electronic and optoelectronic patterning [21, 23, 29, 30]	Post‐growth writing of conducting, doped, thinned, or optically modified regions	Allows selected regions of a 2D material to be programmed without global chemical processing
Ferroic and phase‐memory states [19, 31]	Local switching between stable or metastable structural/ferroic phases, such as laser‐induced In_2_Se_3_ phase conversion	Links optical phase control to nonvolatile memory, ferroic switching, and reconfigurable devices
Chemical and sensing interfaces [22, 32]	Local oxidation, defect creation, or phase‐boundary formation	Creates chemically modified regions or sensing sites by permanent local material modification.

**FIGURE 1:**
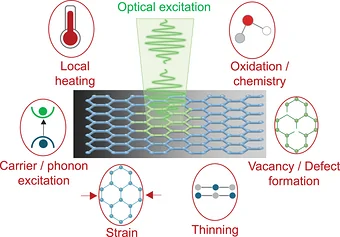
Interacting responses triggered by optical excitation in 2D materials. Optical excitation can generate carrier/phonon excitation, local heating, strain redistribution, vacancy and defect formation, oxidation or other chemical modification, and layer thinning. In atomically thin materials, these responses can occur together and may produce similar optical, Raman, electrical, or topographic signatures. The main challenge is to distinguish the process that initiates the transformation from the process that stabilizes the final configuration.

## 3 Light‐Induced Phase Transitions and Transformations in 2D Materials

A light‐induced material change should be assigned by separating the process initiating the transformation from the process stabilizing the resulting state. Candidate initiating processes include carrier excitation, electron‐phonon coupling, strong electric fields, local heating, strain release, defect formation, and chemical reactions. Stabilization can arise from a different process involving metastable trapping, thermal quenching, substrate‐mediated strain, vacancies, chemical bonding, or combined ambient effects [1, 6, 18]. Table 2 summarizes the practical classification, and Figure 2 provides one experimental illustration for each class. The seven classes serve as overlapping practical categories based on the dominant experimentally supported mechanism. Multiple processes can contribute under realistic conditions, particularly during ambient laser processing.

**TABLE 2:** Experimental criteria for assigning dominant initiation and stabilization mechanisms in light‐induced transformations of 2D and layered materials.

Class	Candidate initiating process	Controlled analysis and quantitative descriptors	Evidence supporting initiation	Evidence for stabilization	Representative examples
1. Carrier‐phonon‐coupled transition	Photoexcited carriers redistribute energy through selective electron‐phonon coupling and modify the ordered state	Absorbed fluence, electron temperature, effective heat capacity, pump‐probe delay, relaxation time	Nonequilibrium electronic response and selective energy transfer differ measurably from equilibrium heating	A long‐lived electronic or lattice state persists after the initial excitation without measurable compositional change	1T‐TaSe_2_ [33] and photoinduced separation of electronic and lattice CDW order in 1T‐TiSe_2_ [36]
2. Strong‐field or THz‐driven transition	Intense THz excitation directly drives an order parameter, collective mode, or lattice strain	Peak THz field, pulse number, excitation spectrum, SHG response, conversion time	The response scales with THz‐field strength, and phase‐sensitive or structural measurements verify the induced state	A metastable electronic or structural state persists after the THz pulse, with compositional verification where relevant	THz‐driven MoTe_2_ [34], 3R‐Ta_1+_ *_x_* Se_2_ [37], and WTe_2_ [38]
3. Thermally dominated transformation	Local temperature rise, heat accumulation, annealing, or quenching initiates conversion	Absorbed power, repetition rate, dwell time, calculated or measured temperature, substrate heat transport	Phase or composition follows temperature, heat accumulation, or cooling conditions across controlled exposures	Persistence is analyzed separately against quench history, vacancies, strain, or other post‐heating changes	Laser‐processed MoTe_2_ with thermally driven conversion [35, 39] and fs‐laser In_2_Se_3_ [31]
4. Strain or substrate‐assisted transition	Optical exposure alters strain, adhesion, wrinkling, delamination, or interfacial contact	Raman strain shift, wrinkle geometry, substrate, adhesion state, exposure dose	Phase formation correlates spatially with strain release or altered flake‐substrate contact	The converted state remains associated with the altered mechanical or interfacial configuration	Laser‐wrinkled In_2_Se_3_ [19] and strain‐controlled persistence of the photoinduced state in 1T‐TaS_2_ [40]
5. Defect or vacancy‐assisted conversion	Optical exposure creates or redistributes defects that favor another structural state	Vacancy density, elemental ratio, accumulated dose, defect‐sensitive microscopy or spectroscopy	Structural conversion correlates with independently identified vacancies or defect populations	Persistent defects modify the energetic balance and support retention of the converted state	Te‐vacancy‐assisted MoTe_2_ [20, 41] and vacancy‐stabilized laser conversion in MoTe_2_ [35]
6. Chemical or oxidation‐assisted transformation	Optical exposure activates reactions involving oxygen, water, residues, or adsorbates	Reactive atmosphere, laser dose, oxygen content, oxidation‐state fraction, chemical spectral intensity	Chemical‐state or compositional signatures evolve with optical exposure and environmental conditions	New bonds, oxidation states, or modified composition remain after illumination	Laser‐oxidized black phosphorus [22], photo‐oxidized monolayer WS_2_ [42], and laser‐transformed MoS_2_/MoO*_x_* [43]
7. Mixed ambient response	Several thermal, mechanical, defect, chemical, or electronic contributions are experimentally detected	Fluence, repetition rate, atmosphere, substrate, scan history, composition, thickness, morphology	Independent observables support more than one active contribution under related processing conditions	Several measured stabilization processes contribute to retention	Examples requiring multi‐process analysis include MoTe_2_ [21, 39] and laser‐thinned/oxidized MoS_2_ [43].

**FIGURE 2:**
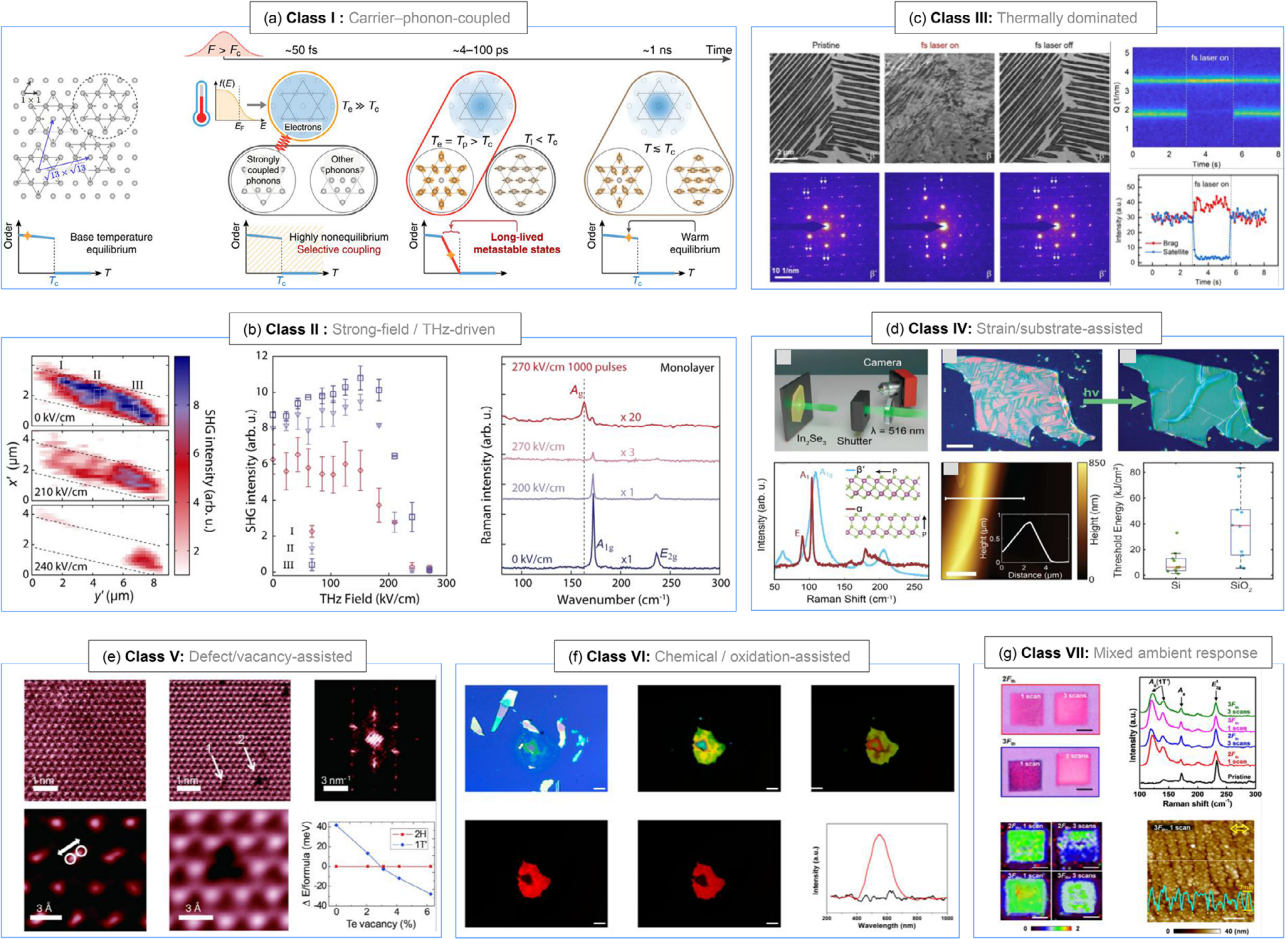
Representative experimental examples of light‐induced phase transitions and transformations in 2D and layered materials. (a) Carrier‐phonon‐coupled transition. Ultrafast excitation of 1T‐TaSe_2_ produces a long‐lived metastable charge‐density‐wave state through mode‐selective electron‐phonon coupling. Adapted from ref. [33], under the terms of the Creative Commons Attribution 4.0 License (CC BY 4.0). Copyright 2019, Science Advances. (b) Strong‐field / THz‐driven transition. In atomically thin MoTe_2_, high‐field THz excitation suppresses the second‐harmonic generation (SHG) response of the non‐centrosymmetric 2H phase and induces Raman signatures of the 1T′ phase. Adapted from ref. [34], under the terms of the Creative Commons Attribution 4.0 License (CC BY 4.0). Copyright 2023, Springer Nature. (c) Thermally dominated transformation. In In_2_Se_3_, femtosecond‐laser irradiation reversibly erases and restores *β′* phase domain structures, with *β′↔β* switching reported as consistent with thermal accumulation. Adapted with permission [31]. Copyright 2025, American Chemical Society. (d) Strain/substrate‐assisted transition. Focused laser exposure of β′‐In_2_Se_3_ induces wrinkling and enables conversion toward the *α* phase through manipulation of accumulated internal strain. Adapted with permission [19]. Copyright 2025, American Chemical Society. (e) Defect/vacancy‐assisted conversion. In MoTe_2_, Te vacancies trigger local 2H→1T′ structural conversion, as shown by atomic‐resolution imaging and calculations. Adapted with permission [20]. Copyright 2015, Science. (f) Chemical / oxidation‐assisted transformation. Ambient laser modification of black phosphorus produces oxidized phosphorene regions that become optically active and exhibit fluorescence, consistent with chemical transformation. Adapted with permission [22]. Copyright 2015, American Chemical Society. (g) Mixed ambient response. Femtosecond‐laser processing of MoTe_2_ produces phase modification, thinning, and surface nanostructuring across distinct fluence regimes accessible within the same laser‐processing platform. Adapted with permission [21]. Copyright 2020, American Chemical Society.

The first two classes involve electronic or field‐driven control of lattice stability. In carrier‐phonon‐coupled transitions, photoexcited carriers modify bonding forces and transfer energy selectively to lattice modes. The long‐lived metastable charge‐density‐wave state in 1T‐TaSe_2_ is a representative example: ultrafast excitation changes the electron distribution, and mode‐selective electron–phonon coupling drives the system into a metastable state distinct from the equilibrium phase [33] (Figure 2a). THz‐driven transitions form a related class in which the applied field provides the controlled external stimulus. In atomically thin MoTe_2_, high‐field THz excitation induces 2H→1T′ conversion without measurable tellurium loss [34] (Figure 2b). Second‐harmonic generation (SHG), Raman spectroscopy, diffraction, and compositional analysis confirm the structural transformation, although the microscopic coupling may involve field‐induced carrier redistribution before lattice conversion. SHG is sensitive to changes in crystal symmetry, especially inversion symmetry, so changes in SHG intensity or polarization can provide a phase‐sensitive marker when competing phases possess different symmetries. SHG evidence gains greater mechanistic value when combined with Raman, diffraction, or other independent structural measurements.

Thermally dominated transformations are governed by local temperature rise, heat accumulation, annealing, quenching, or cooling history. Thermal pathways can be highly useful when the heat dose, repetition rate, substrate, and recovery process are known. Reliable assignment requires evidence that the final phase tracks thermal variables independently of optical‐field strength. Femtosecond‐laser switching in In_2_Se_3_ provides a clear example: the *β′↔β* transition is evidenced by reversible erasing and recovery of domain structures, diffraction changes, and Bragg/satellite intensity evolution, with the observed switching reported as consistent with thermally accumulated excitation [31] (Figure 2c). A clearer separation of thermal initiation and defect stabilization is provided by laser‐heated MoTe_2_, where accumulated heating drives conversion and tellurium vacancies support irreversible retention [35]. The example shows that laser‐driven phase switching can be reproducible even when the controlling variable is heat, provided that the thermal controls support the assigned mechanism.

Strain/substrate‐assisted transitions occur when optical exposure changes the mechanical or interfacial state that controls phase stability. The substrate can act as an active control parameter by imposing strain, pinning domains, altering adhesion, and determining which phase remains after excitation. Laser‐induced wrinkling in In_2_Se_3_ is a representative case. Focused illumination produces wrinkling and enables the *β*′→*α* transition at room temperature by manipulating accumulated internal strain [19].

Defect‐ and chemistry‐assisted transformations are stabilized by changes in local composition or bonding. In defect/vacancy‐assisted conversion, laser exposure creates or redistributes atomic defects that favor a new phase. Laser‐patterned MoTe_2_ homojunctions provide a clear example: Te vacancies trigger the local 2H→1T′ conversion, producing a stable metallic heterophase contact region [20] (Figure 2e). In chemical or oxidation‐assisted transformations, the final state is held by new bonds, oxidation state, functionalization, or adsorbate removal. Ambient laser modification of black phosphorus illustrates the chemical class, where laser exposure produces phosphorene oxide/suboxide regions with distinct optical activity [22] (Figure 2f). These cases can be valuable for contacts, sensing, and permanent patterning, although they require chemical and stoichiometric diagnostics before being assigned to a structural phase transition.

Mixed ambient responses arise when independent measurements support contributions from several processes under related processing conditions. Femtosecond‐laser processing of MoTe_2_ illustrates the difficulty of mechanism assignment because nonlinear imaging, thinning, structural conversion, defect formation, and nanostructuring appear across different fluence regimes [21] (Figure 2g). Distinct processing regimes alone are insufficient evidence for simultaneous mechanisms at a single condition. Assignment to the mixed class requires correlated structural, chemical, thermal, mechanical, or spatial evidence supporting more than one active contribution.

The seven classes describe dominant experimentally supported responses and can overlap under realistic processing conditions. Assignment to a specific class requires systematic dependence on a physically relevant control variable together with independent verification of the resulting material state. A mixed response should be assigned only when independent measurements support contributions from several processes. Cases lacking sufficient evidence for a dominant mechanism should remain unresolved until additional controls separate the competing contributions. Quantitative descriptors serve as material‐specific experimental benchmarks and universal boundaries between mechanistic classes require separate evidence.

## 4 Experimental Identification of Light‐Driven Mechanisms

Assigning a light‐driven transformation requires stronger evidence than persistent optical, electrical, or topographic contrast alone. The experimental problem involves identifying the physical variable responsible for conversion and the process preserving the resulting state after illumination ends. Reliable assignment requires controlled perturbations combined with independent structural, chemical, temporal, and spatial measurements. Figure 3 summarizes an experimental sequence connecting material verification, candidate‐driver analysis, temporal measurements, and stabilization analysis. Single observables can identify material changes without uniquely identifying their microscopic origin. Raman signatures can accompany structural conversion, strain, heating, defects, thickness changes, or chemical modification under different experimental conditions. Optical contrast can likewise arise from several processes producing experimentally similar final images. Mechanistic evidence becomes substantially stronger when phase formation follows a physically relevant control variable, and independent measurements verify the resulting material state.

**FIGURE 3:**
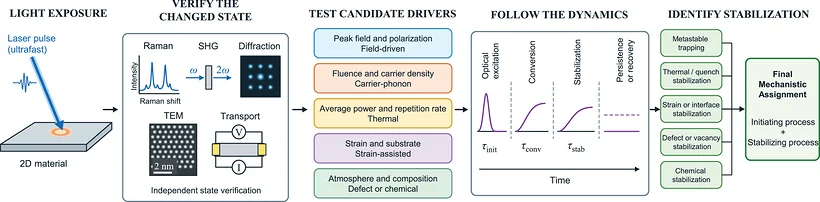
Experimental workflow for assigning initiation and stabilization mechanisms in light‐driven transformations of 2D materials. A changed optical, electrical, or topographic response first requires independent verification of the resulting structural, electronic, or chemical state. Controlled variation of field strength, fluence, repetition rate, temperature, substrate, strain, atmosphere, and composition can then identify experimental variables governing conversion. Time‐resolved measurements separate the initiation time τ_init_, conversion time τ_conv_, and stabilization time τ_stab_ across the evolving material response. Measurements after illumination determine if persistence originates from metastability, thermal history, strain, defects, chemical modification, or overlapping processes. Final mechanistic assignment requires separate identification of the process initiating conversion and the process stabilizing the written phase.

### 4.1 Controlled Perturbations for Mechanism Discrimination

Carrier‐phonon‐coupled transitions require measurements connecting nonequilibrium electronic excitation with subsequent evolution of the ordered material state. Absorbed fluence, electronic temperature, pump‐probe delay, and relaxation time provide useful quantitative variables for comparing excitation conditions. Ultrafast electron calorimetry in 1T‐TaSe_2_ revealed a long‐lived metastable charge‐density‐wave state associated with strongly mode‐selective electron‐phonon coupling [33]. Comparison with equilibrium heating becomes especially valuable because selective energy transfer can produce responses that differ strongly from thermalized lattice excitation. Terahertz‐driven transitions require systematic dependence on the applied electric‐field amplitude and independent verification of structural conversion. Peak field, local field enhancement, pulse number, polarization, and time‐dependent phase‐sensitive signals provide useful experimental descriptors. Atomically thin MoTe_2_ shows a reproducible decrease of second‐harmonic generation above a free‐space THz field near 183 kV cm^−^^1^, corresponding to an estimated enhanced field near 4 MV cm^−^^1^ [34]. Structural and compositional measurements subsequently confirm formation of intrinsic 1T′‐MoTe_2_ without measurable tellurium loss. The microscopic coupling remains open, with THz‐driven carrier redistribution and charge doping discussed as possible routes toward lowering the structural barrier.

Thermally dominated transformations should be evaluated using variables governing temperature rise, energy accumulation, and heat removal. Laser power, irradiation duration, repetition rate, base temperature, substrate thermal transport, and cooling conditions provide useful experimental controls. Few‐layer MoTe_2_ provides a particularly clear example because accumulated heating was identified as the main driver of laser‐induced 2H→1T′ conversion [35]. Te vacancies generated during local heating were associated with irreversible retention of the converted 1T′ phase. The same experiment demonstrates why initiation and stabilization mechanisms should be examined separately. Encapsulation provides an additional thermal and environmental control for laser‐processed MoTe_2_ under repeated optical exposure. Air‐exposed or single‐side‐passivated flakes can form tellurium or Mo_3_Te_4_ through accumulated heating and atomic evaporation. Hexagonal‐boron‐nitride encapsulation suppresses evaporation and allows stable 1T′ patterning under suitable irradiation conditions [39]. Such comparisons show how heat dissipation and material confinement can alter the final product under nominally related optical exposure.

Strain‐assisted transformations require direct correlation between structural conversion and measured mechanical or interfacial changes. Substrate choice, initial strain, wrinkle formation, delamination, adhesion, and Raman strain markers provide experimentally accessible control variables. Laser‐induced wrinkling in *β*′‐In_2_Se_3_ enables room‐temperature conversion toward the *α* phase through manipulation of accumulated internal strain [19]. AFM, Raman measurements, and spatial phase mapping can connect the modified mechanical configuration with the observed structural conversion. Defect‐assisted conversion requires independent measurement of vacancies, stoichiometry, defect redistribution, or defect‐sensitive structural signatures. Laser‐patterned MoTe_2_ provides a direct example because atomic‐resolution imaging and calculations identified tellurium vacancies as triggers for local 2H→1T′ conversion [20]. Later work showed that local vacancy ordering can nucleate 1T′ regions and that photoexcited carriers can accelerate vacancy diffusion [41]. Such results also show that mechanistic classes can overlap when electronic excitation controls the motion of structural defects.

Chemical transformations require direct evidence of altered bonding, oxidation state, or elemental composition after optical exposure. Atmosphere, oxygen availability, humidity, irradiation dose, and chemical spectroscopy provide especially useful controls under ambient processing conditions. Laser treatment of black phosphorus produces phosphorene oxides and suboxides whose oxidation level depends systematically on laser power [22]. Some modified regions remain stable under ambient conditions for approximately two weeks, linking permanent optical changes with persistent chemical modification. Mixed responses require stronger evidence because several mechanisms can generate similar final observables under ambient processing conditions. Assignment to the mixed class should require independent measurements supporting contributions from several active processes during related exposure conditions. Distinct fluence regimes alone are insufficient evidence for simultaneous microscopic mechanisms. Femtosecond processing of MoTe_2_ provides a clear example of this experimental caution because nonlinear imaging, layer removal, structural conversion, and nanoripple formation appear across different fluence ranges [21].

### 4.2 Temporal Signatures of Initiation and Stabilization

Temporal measurements provide an independent route for separating optical excitation from later conversion and stabilization processes. Three descriptive times can organize experimental observations without implying universal kinetic constants across different material systems. The initiation time τ_init_ describes the earliest measurable response connected with optical excitation and energy transfer. The conversion time τ_conv_ describes development of the experimentally identified structural, electronic, mechanical, or chemical state. The stabilization time τ_stab_ describes formation of the persistent or recovered configuration following the conversion process. Large differences among these times can expose sequential processes hidden in post‐exposure measurements. Femtosecond excitation can generate carriers or coherent lattice motion long before slower structural rearrangement, heat transport, defect migration, strain relaxation, or chemical reaction reaches completion. Time‐resolved diffraction, photoemission, transient optical spectroscopy, Raman measurements, SHG, and electrical probes can provide complementary information when suitable temporal resolution is available. Longer measurements after illumination remain equally valuable because persistence or recovery helps identify the mechanism responsible for retention.

THz‐driven MoTe_2_ provides a particularly clear demonstration of separated excitation and conversion times. The SHG response reaches approximately twice its initial value near 20 ps before decreasing strongly at later delays [34]. The signal falls to roughly twenty percent of its initial value near 12.5 ns and disappears at longer delays. Single‐shot measurements identify a characteristic conversion time near 10 ns for leaving the original 2H polymorph. Measurements near one minute confirm persistence of the resulting metastable state after the original THz excitation has vanished. The early SHG increase was interpreted as possible nucleation of a transient lower‐symmetry structure requiring additional structural verification. Such behavior illustrates an important limitation of assigning transient intermediates from a single spectroscopic observable. Temporal resolution becomes most informative when intermediate signals are linked with independent structural or electronic measurements.

### 4.3 Quantitative Descriptors and Strength of Mechanistic Evidence

Quantitative descriptors gain mechanistic value when a measured material response follows a controlled physical variable reproducibly. Peak electric field provides an appropriate descriptor for strong‐field excitation under a calibrated enhancement geometry. Absorbed power, repetition rate, estimated temperature rise, and thermal relaxation provide corresponding tests for heating‐driven responses. Strain magnitude, vacancy concentration, chemical‐state fraction, phase fraction, and relaxation lifetime provide useful descriptors for mechanical, defect‐mediated, chemical, and metastable behavior. Absolute thresholds remain specific to material thickness, substrate, atmosphere, geometry, and excitation conditions. A field threshold measured in MoTe_2_ cannot define strong‐field conversion across unrelated 2D material systems. The same limitation applies to critical temperatures, strain values, vacancy densities, and chemical conversion fractions. Quantitative descriptors become most useful when reproducible scaling trends are measured within the same material and experimental geometry.

Mechanistic evidence can be arranged according to increasing experimental strength across four connected levels. Initial evidence documents reproducible optical, electrical, spectroscopic, or topographic modification following controlled exposure. Independent measurements then verify the structural, electronic, ferroic, or chemical identity of the modified state. Controlled perturbations and time‐resolved measurements subsequently connect phase formation with candidate physical drivers. Measurements after excitation finally identify the process responsible for persistence, recovery, or continued evolution of the modified state. Such an experimental sequence converts mechanism assignment from interpretation of post‐exposure images into a set of controlled measurements. The approach also provides a natural basis for near‐field experiments where excitation position can be confined to nanometer dimensions. Spatial localization can reduce uncertainty concerning the initiation site, although structural, chemical, temporal, and environmental measurements remain essential for assigning the resulting material state.

## 5 Controlled Conditions and Real Environments

Controlled experimental conditions are essential for isolating the intrinsic response of a photoexcited 2D material. Cryogenic temperature, ultra‐high vacuum, encapsulation, and ultrafast probes reduce active variables and help separate electronic, phononic, and structural responses [1, 44]. Such experiments provide a reference response against which ambient phase writing can be evaluated. Ambient processing introduces substrate adhesion, heat flow, oxygen, moisture, residues, wrinkles, defects, and exposure history as additional control variables [6, 18, 26, 45, 46]. These variables can shift conversion thresholds and change the process that preserves the written state after illumination. Environmental factors can also become deliberate control parameters when their role in conversion or stabilization is supported experimentally. Controlled experiments identify a reference material response, and ambient experiments determine how that response changes in practical device geometries. Reproducibility ultimately depends on identifying the variables governing initiation and stabilization under the chosen experimental conditions. Under ambient far‐field illumination, several active variables can occupy the same irradiated region, creating spatially heterogeneous responses that motivate the next section [18, 21].

## 6 Ambient Far‐Field Laser Phase Engineering

Far‐field laser irradiation remains a powerful method for phase engineering, patterning, thinning, oxidation, defect modification, and device fabrication in 2D materials [17, 18, 23, 46]. Wavelength, pulse duration, repetition rate, fluence, polarization, exposure time, and scan speed provide flexible control over the delivered optical dose. The main limitation for mechanistic assignment is spatial averaging, since a diffraction‐limited spot can contain nanoscale variations in thickness, strain, defects, residues, adsorbates, and substrate contact [18, 21, 23]. Local environments can cross different conversion thresholds during the same exposure and produce different microscopic responses inside one written region.

Femtosecond processing of MoTe_2_ illustrates fluence‐dependent complexity particularly clearly. Low fluence permits nonlinear optical probing without permanent modification, and exposure near the ablation threshold can remove individual layers with nanometre‐scale control [21]. Higher fluence produces structural phase conversion, and controlled excitation can generate subwavelength nanoripples. These regimes show that one optical platform can access probing, thinning, structural conversion, and nanostructuring under different exposure conditions. The resulting state depends on fluence, scan number, repetition rate, substrate heat flow, and the initial condition of the flake. Spatial averaging limits identification of the local nucleation site and the process stabilizing the local modification. Correlated structural, chemical, optical, and spatial measurements remain necessary for assigning the local mechanism [18, 20, 21, 23, 46]. Near‐field excitation offers a complementary geometry by confining the optical field near a sharp tip or nanoscale antenna, reducing spatial averaging and bringing optical writing closer to nanoscale readout [47, 48, 49].

## 7 Near‐Field Excitation for Ambient Nanoscale Phase Control

Figure 4 summarizes the change from diffraction‐limited far‐field illumination to tip‐confined excitation. Near‐field excitation concentrates the optical field near a sharp tip or nanoscale antenna [50, 51, 52], defining the likely initiation region more precisely than a diffraction‐limited spot. For conventional s‐SNOM probes, the lateral near‐field interaction is typically confined to approximately 10–30 nm, with the characteristic scale set mainly by tip‐apex radius and tip‐sample coupling [50, 51, 52]. Modern s‐SNOM routinely reaches optical spatial resolution near 10 nm [50, 52]. The interaction region must be distinguished from the dimensions of any permanent written feature produced after excitation. Such confinement provides a geometry where local nucleation, modification, and nanoscale diagnosis can be connected [49, 52, 53].

**FIGURE 4:**
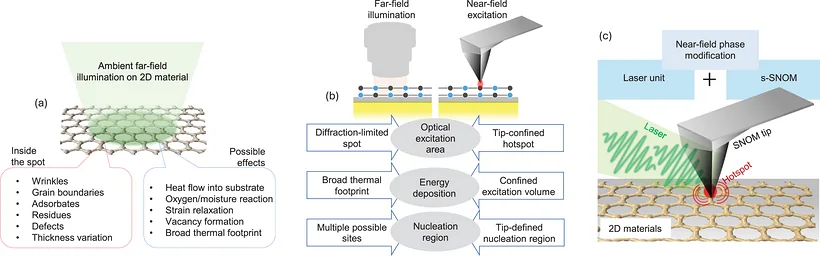
From ambient far‐field exposure to tip‐enhanced near‐field phase control. (a) Under ambient far‐field laser exposure, the irradiated region can contain wrinkles, grain boundaries, adsorbates, residues, defects, and thickness variations. The observed modification may include contributions from substrate heat flow, oxygen or moisture reactions, strain relaxation, vacancy formation, and a broad thermal footprint. (b) Far‐field illumination deposits energy over a diffraction‐limited region, and near‐field excitation confines the optical hotspot near a sharp tip. Such geometry can reduce spatial averaging and define the region where phase conversion is most likely to start. (c) Tip‐enhanced femtosecond excitation provides a route for ambient nanoscale phase modification of 2D materials by combining local optical writing with AFM/s‐SNOM‐based readout and subsequent structural, chemical, and optical verification.

In a tip‐enhanced geometry, the metallic probe acts as a local optical antenna that concentrates incident light near the apex [50, 51, 52]. The same s‐SNOM platform can revisit the modified region with nanoscale topographic and optical readout [52, 53], linking excitation and diagnosis within a common probe geometry. Near‐field optical nanopatterning of graphene provides a useful experimental proof‐of‐principle for this geometry [49]. In that work, femtosecond excitation localized at an s‐SNOM tip produced permanent graphene modifications with reported lateral dimensions of 10–30 nm and sub‐5 nm vertical changes. These dimensions describe the resulting material features. The optical near‐field hotspot requires separate measurement. The written structures evolved from nanoblister‐like features to nanopunch holes as the laser exposure time increased. AFM and s‐SNOM resolved the local topography and optical contrast, and nano‐FTIR detected oxygen‐related vibrational signatures in the modified regions. The study also compared the laser‐pulse interval with the thermal diffusion time in graphene, supporting two‐photon photochemical oxidation as the dominant process.

The graphene study provides an experimental precedent because it combines ambient nanoscale writing with local topographic, optical, and chemical verification [49]. Applying the same geometry to polymorphic TMDs, In_2_Se_3_, charge‐density‐wave compounds, or layered ferroics could define the initiation region locally and test the written phase through phase‐sensitive measurements. Near‐field phase‐control experiments require controls that isolate probe‐specific effects and connect the written region with the hotspot position. Tip‐present and tip‐absent measurements, laser‐on and laser‐off measurements, pulse‐energy series, exposure‐time series, atmosphere control, and substrate comparison should accompany AFM and near‐field optical mapping. Raman phase signatures, symmetry‐sensitive SHG changes, nano‐FTIR, XPS, EELS, EDX, electron diffraction, TEM, or STEM can then verify the structural or chemical identity of the written region when experimentally accessible. Near‐field excitation changes the experimental question from a spatially averaged response to the behavior of a selected nanoscale region. The excitation site can be revisited and compared with local optical, structural, chemical, and topographic measurements. Remaining challenges include minimum writable volume, field‐enhanced heating, probe‐induced mechanical effects, ambient chemistry, and reporting standards that permit comparison among experiments.

## 8 Open Questions and Outlook

Near‐field phase writing now requires quantitative links between the optical interaction region and the material state that survives after excitation. Four experimental questions are especially important for achieving reproducible nanoscale phase control under ambient conditions. The first unresolved question concerns the minimum stable volume that can remain after local optical writing. Conventional s‐SNOM can confine the optical interaction to a few tens of nanometres, yet the final domain may be governed by critical nucleus size, domain‐wall energy, heat diffusion, strain propagation, or defect motion. Future experiments should measure the optical near‐field scale and the resulting structural, chemical, and functional domain size independently.

The second question concerns separation of field‐enhanced excitation from local heating near the probe apex. Femtosecond excitation can limit thermal spreading, although high repetition rate, long dwell time, strong absorption, or weak substrate heat transport can still produce cumulative temperature rise. Near‐field studies should combine pulse‐energy and repetition‐rate scaling with thermal modelling under matched tip and substrate conditions. The third question concerns the physical role of the probe during the writing process. The metallic tip acts simultaneously as an optical antenna, mechanical contact, and nanoscale electromagnetic boundary condition. Tip radius, coating, contact force, tapping amplitude, illumination geometry, tip‐sample distance, degradation, and contamination can each modify the local conversion threshold. Reporting these parameters is essential for comparing phase‐writing results across different instruments and laboratories.

The fourth question concerns selection of materials suited to local optical phase control. Promising systems include polymorphic TMDs, In_2_Se_3_ and related ferroic materials, charge‐density‐wave compounds, and layered topological materials with competing states separated by accessible energy scales. Suitable candidates should combine manageable chemical stability, measurable conversion thresholds, clear structural or spectroscopic markers, and a written state with controlled retention or reset. Progress will depend on experiments that connect local excitation, independent state verification, and retention within the same nanoscale region.

## 9 Conclusions

Current research on light‐driven phase transitions and transformations in 2D materials has progressed from demonstrations of optical modification toward mechanistic assignment and controlled retention. The main challenge is identifying the process that initiates conversion and the mechanism that preserves the resulting phase or modification after excitation. Carrier excitation, phonon coupling, heating, strain, defects, chemistry, and substrate interactions can contribute during one experiment. Reliable assignment requires controlled perturbations, independent material verification, temporal measurements, and material‐specific quantitative descriptors. Far‐field laser processing remains valuable for scalable writing and device fabrication, although diffraction‐limited illumination can average over nanoscale heterogeneity. Tip‐enhanced femtosecond excitation offers a complementary route by localizing optical excitation to the near‐field interaction region and enabling nanoscale readout of the written region. Progress toward reproducible ambient nanoscale phase control will depend on linking local excitation, verified phase identity, and controlled retention locally.

## Author Contributions

**Gour Mohan Das**: Conceptualization; writing – original draft; review and editing. **Mika Pettersson**: Conceptualization; resources; project administration; funding acquisition; supervision; writing – review and editing. All authors approved the final manuscript.

## Funding

The authors acknowledge the support of the Research Council of Finland (project number: 357130).

## Conflicts of Interest

The authors declare no conflicts of interest.

## Data Availability

Data sharing is not applicable to this article as no new data were created or analyzed in this study.
